# Comparing Clothing-Mounted Sensors with Wearable Sensors for Movement Analysis and Activity Classification

**DOI:** 10.3390/s20010082

**Published:** 2019-12-21

**Authors:** Udeni Jayasinghe, William S. Harwin, Faustina Hwang

**Affiliations:** Biomedical Engineering, School of Biological Sciences, University of Reading, Reading RG6 6AY, UK; w.s.harwin@reading.ac.uk (W.S.H.); f.hwang@reading.ac.uk (F.H.)

**Keywords:** actigraph, body worn sensors, clothing sensors, cross correlation analysis, healthcare movement sensing, wearable devices

## Abstract

Inertial sensors are a useful instrument for long term monitoring in healthcare. In many cases, inertial sensor devices can be worn as an accessory or integrated into smart textiles. In some situations, it may be beneficial to have data from multiple inertial sensors, rather than relying on a single worn sensor, since this may increase the accuracy of the analysis and better tolerate sensor errors. Integrating multiple sensors into clothing improves the feasibility and practicality of wearing multiple devices every day, in approximately the same location, with less likelihood of incorrect sensor orientation. To facilitate this, the current work investigates the consequences of attaching lightweight sensors to loose clothes. The intention of this paper is to discuss how data from these clothing sensors compare with similarly placed body worn sensors, with additional consideration of the resulting effects on activity recognition. This study compares the similarity between the two signals (body worn and clothing), collected from three different clothing types (slacks, pencil skirt and loose frock), across multiple daily activities (walking, running, sitting, and riding a bus) by calculating correlation coefficients for each sensor pair. Even though the two data streams are clearly different from each other, the results indicate that there is good potential of achieving high classification accuracy when using inertial sensors in clothing.

## 1. Introduction

In many countries, a significant increase can be seen in the number and proportion of older adults year on year. The population of people over 60 years old is projected to increase in Europe, Northern and Latin America, Asia and Africa from the year 2015 to 2030 [1]. The number of people who have noncommunicable diseases is also projected to increase significantly by 2030 [2]. Generally older people are more prone to noncommunicable diseases [2] resulting in high care costs in each country. In OECD (Organisation for Economic Co-operation and Development) countries, an annual increment of 4.8% of the cost allocated for long-term monitoring from 2005 to 2011 was seen. It is predicted that this cost will double in the period from 2015 to 2060 [3].

Home-based monitoring potentially offers a cost-effective mechanism for prevention of disease and promotion of healthier lifestyles. A number of factors have to be taken into account when using a long-term monitoring system, such as whether these systems are reliable for measuring real time data, are safe to use with patients, have high power efficiency, and provide clinically useful data. Wearable sensors have the capability to provide efficient monitoring of daily routines for a long period in a cost effective way [4].

A growing interest in health monitoring has led to the commercial availability of a number of wearable sensors for self-monitoring. Consumer products for self-monitoring generally comprise a single device, often wrist worn, which may hinder the accuracy of the data analysis and classification. In contrast, in research work, multiple sensor devices are often used in order to achieve a higher accuracy in activity classification. However, there are feasibility issues with the wearing of multiple sensors on a daily basis in a residential environment. There are also challenges in maintaining a consistent sensor orientation and approximate location with respect to the body during the data collection periods. Further, in healthcare the patient or research participant may not have the patience, or abilities to attach multiple sensors each day. Embedding sensors into the clothing may, to some extent, address both issues of wearing multiple sensors every day and managing the sensor orientation and approximate location.

This study considers the quality of data that would arise from inertial sensors embedded into clothes that people wear on a daily basis.

It examines whether these sensor devices would be able to provide data as accurate as that collected by sensors attached to the person. In particular, can the data be used to predict the actions and behaviour of the individual and allow activity classification?

The aim of this research is to investigate and quantify to what extent the data obtained from the clothing sensors can be used in characterising activities, as compared with body worn sensor data. To achieve this, sensor data were collected from body worn sensors and sensors attached to three different clothing types, across a range of daily activities. The correlation coefficients were calculated between the clothing-embedded and worn data to check how much they agree with each other across a range of daily activities and different styles of clothes.

## 2. Related Work

Research relating to the use of wearable sensors with older adults has largely been in three areas – indoor tracking, activity classification and real-time vital sign monitoring [5]. Activity classification using body worn inertial sensor data in long-term monitoring is a well-established approach [6]. Accelerometers are being used as the key instrument, while gyroscopes and barometric pressure sensors are also used in some studies. Out of those studies some are using a single sensor while others are using multiple sensors for activity recognition. For example, a single sensor, i.e., a sensor only on the waist, thigh, lower-back and thigh, in activity classification of the whole body can be seen respectively in [7,8,9,10]. Other studies, using multiple sensors, investigate the accuracy of activity classification compared across placement of the sensors on the wrist, hip, neck, knee, chest, lower arm, lower back, upper arm and ankle. Montoye et al. [11] observed high accuracy in activity classification for three levels of physical activities, i.e., SB (sedentary behaviour), LPA (light-intensity physical activity) and MVPA (moderate-to vigorous-intensity physical activity) based on thigh data, high accuracy in classifying SB based on (non-dominant) wrist data, and low accuracy in classifying physical activities based on (dominant) wrist and hip data. Hence, they concluded that it is better to use thigh data or non-dominant wrist data in analysing different levels of physical activities. Cleland et al. [12] found that, of chest, wrist, lower back, hip, thigh and foot sensor data, hip data scored the highest accuracy in activity classification. However, they [12] also noted that further studies should be carried out in order to find the optimal sensor placement across multiple activities, since their study focused only on activities such as walking, lying and sitting. As both upper body and lower body movements contribute to locomotion [13], it is better to investigate movements from both sides of the body, rather than just one side.

Analysis of above mentioned sensor data related to activities may seek to find patterns of activities or movement quality. In most of the studies, pattern recognition algorithms were used in activity classification, such as decision trees ([10,14,15]), KNN (k-nearest neighbours algorithm) ([15,16,17]), SVM (Support Vector Machine) ([9,18,19]) and other algorithms (C4.5, RF (Random Forest), NB (Naive Bayes), Bayesian).

Even though there are numerous research studies on activity classification with sensor data, very few have been conducted on sensors attached to everyday clothes. One study highlighted that there was little to no research validating the measurements of IMUs (Inertial Measurement Unit) attached to loose clothes [20]. Their research aimed to validate the temporal motion from the sensors attached to the clothes. As the clothes, a tight fitting vest and a tight jacket were used. Their main intention was to validate the sensor readings by calculating four parameters, i.e., raw error, standardised error (Cohen scale), Pearson’s correlation and mean difference. Five inertial sensor devices (weighing 23 g, with dimensions 55 mm × 30 mm × 13 mm) were used, where two were strapped onto the Cervical vertebrae segment(C7) and Thoracic vertebrae segment (T12), one was placed on a jacket at C7, and the other two sensors were sewn into two pockets of a tight fitting elastic heart rate monitor vest so that they were posterior to the C7 and T12 sensors. The study focused on only one activity, that is, dead-lifting. When comparing the raw error, Cohen scale, correlation and mean difference of the data sets, only the anterior-posterior acceleration was used. They were able to see a high similarity between the sensor values that were obtained from both mechanisms, owing to the single activity that they conducted with the tight clothes.

A second research study reported that sensors mounted onto clothes, instead of strapping them onto a structure with rigid bands, gives a better signal variation so that it may make the activity recognition procedure easier [21]. For their data collection, a pendulum and three different fabric materials (denim, jersey and roma) and three tri-axial accelerometers were used. The fabric was attached to the end of the pendulum and three accelerometers attached such that one was at the tip of the pendulum (fixed in place with a rigid band), a second one was in the middle of the fabric, and a third was at the edge of the fabric. After attaching the calibrated sensors, the pendulum was released from a horizontal position and data was collected for 10 seconds. The experiment was done with and without an additional weight at the end of the pendulum. The Euclidean distance and one-way analysis of variance were calculated when calculating the similarity of the signals (data from sensors attached with rigid bands as compared with sensors attached to different fabric materials). The objective was to predict whether the pendulum was swinging with or without a weight attached to the end. For this prediction, SVM and DRM (Discriminative Regression Machines) were used. The conclusion of their research work was that the fabric’s nature of deforming movements in various directions makes it easier to predict the motion, compared with the sensor data obtained from the sensors attached with the rigid bands.

Hence it can be concluded that more information is needed to assess the true value of embedding sensors into clothing to allow better representation of human movement and activity classification.

## 3. Materials and Methodology

The aim of the present study is to compare and contrast how clothing sensor data patterns correlate/deviate from body worn sensor data, across three different types of clothing.

### 3.1. Data Collection Procedure

Data were collected from one participant (the first author) over three normal working days. On each day, the participant wore a different type of clothing (loose slacks, pencil skirt, and frock/knee-length dress), and multiple sensors were worn in pairs on the clothing and the body. The sensors and their placement are described further in the next section. An activity log was kept and used to annotate the data files. The main activities were walking, running, sitting as well as other daily activities including riding on a bus.

### 3.2. Sensor Placement

**Figure 1:**
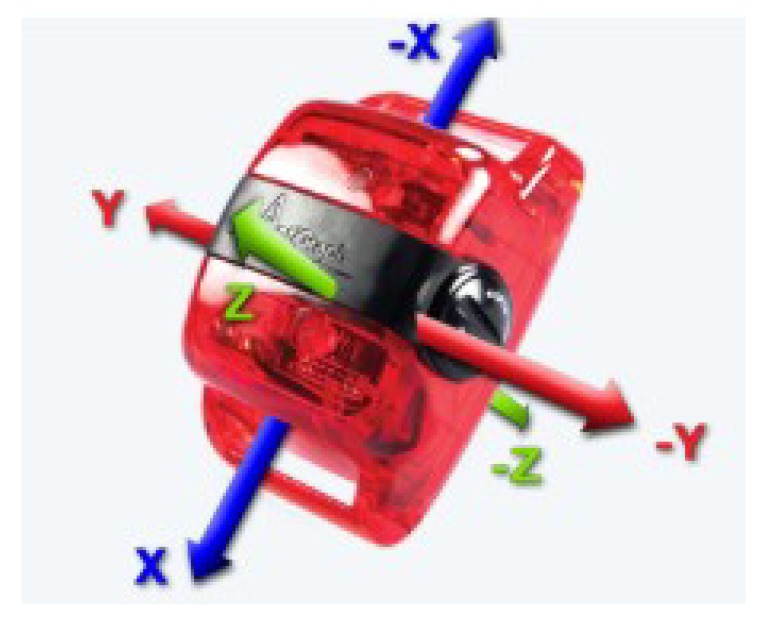
Coordinate frame of the Actigraph device. (Image from Actigraph website https://www.actigraphcorp.com).

**Figure 2:**
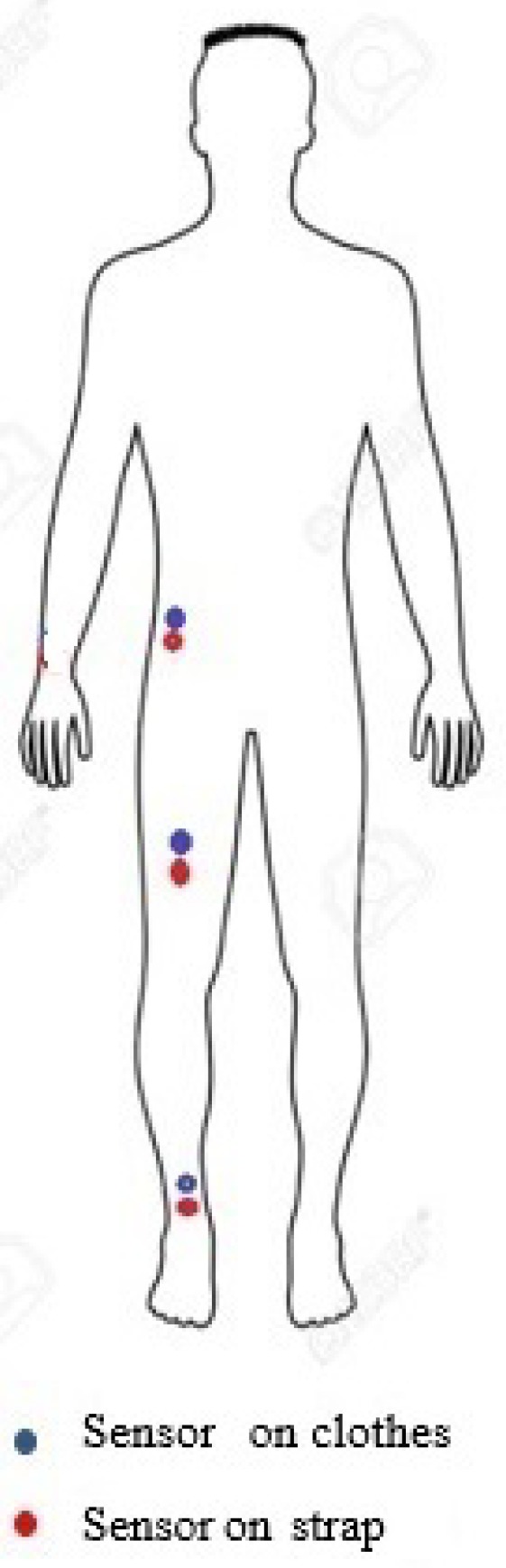
Sensor placement on subject and on subject’s clothes.

Actigraph tri-axial accelerometers (wGT3X-BT, weighing 19 g and measuring 4.6 cm×3.3 cm×1.5 cm, as shown in Figure 1) were worn in pairs, such that one sensor was strapped onto the body and the other was sewn to the clothes in a similar location to the body-worn sensor. As the optimal places to mount sensors are not yet well defined [12], we mounted one sensor pair on the waist to track upper body movements, and two other sensor pairs on the upper thigh and ankle to track lower body movements [13]. Hence, sensor pairs were placed at the participant’s waist and upper-thigh for the pencil skirt (41 cm long, with a 38 cm inch perimeter at the thigh) and the frock (48 cm inch perimeter at the thigh). For loose slacks, a further pair of sensors was worn on the ankle and hem of the slacks. The body worn sensors were always placed just below the sensors on the clothes, as shown in Figure 2. The participant was 152 cm in height, and wore UK women’s size 6 clothes. The orientation of the sensors was set such that the *y*-axis was aligned most closely to the axis of acceleration from gravity. Table 1 shows the duration of data collection, type of clothing and sensor placement.

The sensor devices were initialised with the Actilife (https://www.actigraphcorp.com/support/software/actilife/) software to synchronise their internal clocks. Additionally, at the start of each day of data collection, the participant performed a jump in order to create a distinctive marker in the accelerometry data that could be used to further check the synchronisation. Furthermore, each pair of sensors (one in clothes and one on the body) were tapped synchronously four times to ensure data from sensor pairs could be time-aligned. At the end of each data collection period, another jump was performed to identify the point where the data collection was completed, and provide an indication of any potential sensor time drift.

### 3.3. Data Analysis

The data were analysed in terms of sensor pairs, in order to compare the body worn with the clothing worn data. Comparisons were also made across different activities and the different clothing types. The data were analysed in MATLAB.

#### 3.3.1. Preprocessing the Data

The data from both sensors in a pair were first time-aligned, based on the “jump” and the “tap” markers. Next, the time lag between the two sets of sensor readings for each activity was estimated using a cross correlation, because there can be time lags between the body-worn and the clothing-mounted sensor readings owing to factors such as the stiffness of clothing material (which causes swing) and cloth dynamics for each activity. The maximum cross correlation value was then used to determine the lag between the two signals, and this lag was adjusted in order to bring the two signals into alignment.

Secondly, an orientation correction was applied to both sets of data. When attaching the sensors onto the body and to the clothes, there may be discrepancies in the orientations between the two sensors in a pair. Hence in order to maintain a reasonably similar orientation for each sensor pair, each data set was rotated along a common axis so as to align the principal direction of gravity with the *y*-axis of the sensor. This correction can be computed easily using Rodrigues’ rotation formula [22] and identifying the axis of rotation as being perpendicular to both the gravity vector and the *y*-axis, and the rotation about this axis is therefore the angle between these two vectors. Data where this rotational correction has been applied is termed the ‘rotated data set’.

These preprocessing techniques were carried out in order that the data from the two sensors in each pair could be meaningfully compared.

#### 3.3.2. Activity Extraction

Using the activity log, data segments corresponding to four activities (walking, running, sitting, bus ride) were extracted for each day/clothing type. From these segments, three shorter instances (30–40 s/1500–2000 data points) of each activity were identified and extracted for further analysis.

#### 3.3.3. Comparing the Similarity of the Body-Worn and Clothing-Mounted Sensors

After establishing the normality of the data [23], Pearson’s correlation coefficient was calculated for each sensor pair to assess the strength of the linear relationship between the two signals [24].

#### 3.3.4. Activity Classification

We also wished to investigate the possibility of using the clothing sensor data in activity classification as productively as the body worn sensors. For this purpose, the data were categorised into four classes: walking/running, transition of a movement, sitting and standing. The analysis examined only the ’thigh’ sensor data. When a subject is sitting, the thigh is often in a perpendicular posture with respect to the standing posture, hence sitting and standing would be more easily distinguished with thigh sensor orientation data as compared with waist or ankle sensor data.

Furthermore, the *y*-axis accelerations (gravity axis) were used for the classification, because this axis exhibited the most noticeable differences across activities in acceleration values. When a subject is standing, the gravity axis acceleration is (following alignment) close to the *y*-axis value. When the subject is sitting, the *y*-axis is now perpendicular to the gravity vector so values are close to zero. When the subject is moving, the *y*-axis values are changing significantly based on the additional accelerations that result from these movements.

The features used for classification were chosen to emphasise information about posture and movement, including movement transitions. Transitions include sit-to-stand/stand-to-sit activities which would cause the *y*-axis acceleration to increase/decrease suddenly, sit-to-walk/run could again increase the acceleration suddenly, and walk/run-to-stand would cause a sudden reduction of the acceleration. Two features were used in this classification. To track postural changes, the *y*-axis acceleration values were used, while the moving variance of the *y*-axis acceleration values was calculated to track these transitions. A window size of 250 milliseconds was chosen to ensure that even the acceleration changes in short periods were captured.

A decision tree was implemented to classify the data into activities by defining threshold values, based on visual inspection, for the *y*-axis (gravity) acceleration and the *y*-axis moving variance values. Threshold values were estimated for both body-worn and clothing-mounted sensor data.

Both body worn and clothing data files were then classified into activities by using the decision tree. Finally, a confusion matrix was created to observe how the classification outputs differed from body worn data and clothing sensor data, by considering the classifications of body worn sensor data as the benchmark data set.

## 4. Results

### 4.1. Activity-Wise Time-Alignment

**Figure 3:**
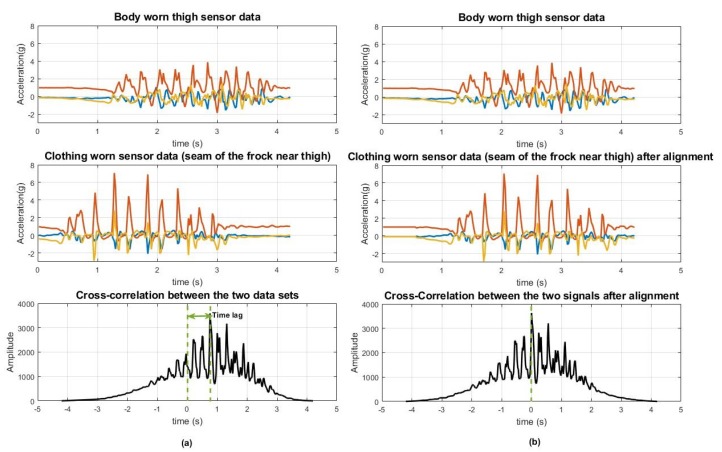
(**a**): Left side 3 plots: Tracked time lag between body worn and clothing sensor data for running when the subject was wearing a frock, (**b**) Right side 3 plots: Signals after the alignment using cross-correlation value. (**b**) After alignment, maximum cross correlation was observed at 0 s.

Figure 3 illustrates the cross correlation values plotted over time for one of the running data segments. The point at which the cross correlation reaches a maximum value indicates the lag between the two signals. The graph shows Day 3 (Frock) running data from the thigh sensor, and for this specific activity, the lag was 38 data points (approximately 0.76 s delay).

After adjusting for the delay based on the cross correlation maximum value, the time-aligned signals are as shown in the right-hand plots in Figure 3, with a maximum cross-correlation now appearing at 0 s, indicating that the delay between the two signals was minimised after applying this technique. When the correlation coefficient is calculated without considering this time lag, for this running instance, the value was 0.4136 and after the lag was corrected the correlation coefficient value was 0.6345. Likewise, the time lag between body worn and clothing worn data set for each activity segment was calculated and corrected before examining the correlation coefficient values for each activity.

### 4.2. Descriptive Analysis of Acceleration Data

**Figure 4:**
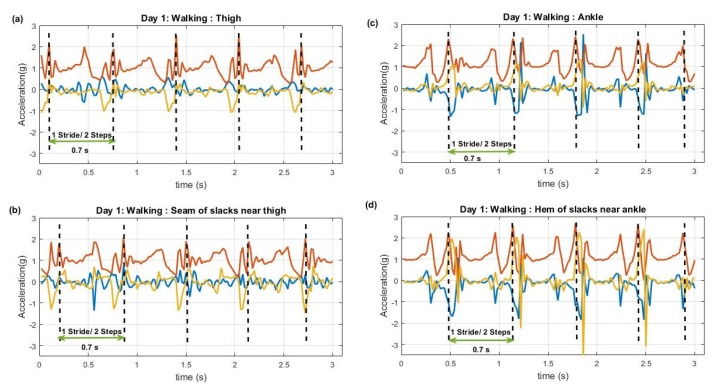
Walking from Day 1 (slacks). (**a**): Data from thigh worn sensor, (**b**): Data from seams of slacks near thigh, (**c**): Data from ankle worn sensor, (**d**): Data from hem of slacks near ankle. Red axis: vertical acceleration, Blue axis: anterior-posterior acceleration, yellow axis: mediolateral acceleration. Note the similarity of signals between clothing and body worn sensors for walking

Figure 4 illustrates walking data extracted from thigh and ankle sensor pairs when the subject was wearing slacks. The sensors were on the right leg, thus two peaks can be interpreted as a single stride (2 steps) as indicated. According to the data it was calculated that typical stride (two steps) time here was approximately 0.7 s.

**Figure 5:**
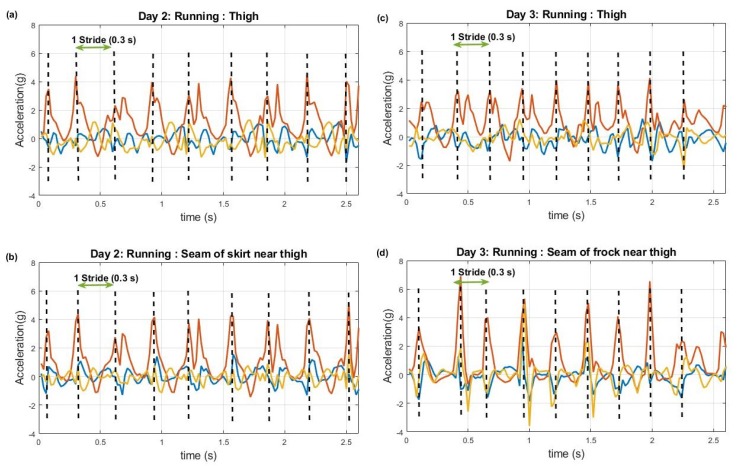
Running data from Day 2 (Skirt; left graphs) and Day 3 (Frock; right graphs). (**a**): Day 2 data from thigh, (**b**): Day 2 data from seams of skirt near thigh, (**c**): Day 3 data from thigh, (**d**): Day 3 data from seams of frock near thigh. Red axis: vertical acceleration, Blue axis: anterior-posterior acceleration, yellow axis: mediolateral acceleration. Note the similarity of signals between clothing and body worn sensors for skirt data verses the high accelerations present in frock data.

Figure 5 shows running data from the sensor pairs that were on (and over) the thigh when the subject was wearing a pencil skirt (left graphs) and frock (right graphs) respectively. According to these data it can be seen that typical stride time (two steps) for running was approximately 0.3 s. Even though the acceleration values of pencil skirt data have relatively similar values with body worn sensor data, the frock data in contrast comprise higher acceleration values with sharp peaks when compared to body worn data.

### 4.3. Correlation Coefficient Value Analysis

When examining the correlation coefficient values, five different sets of data were compared to determine from which data set the maximum correlation coefficient could be found. The five different data sets were the original data set, the time aligned data set, rotated data along the gravity axis, time-aligned and rotated data and finally the time-aligned, rotated and activity wise time-aligned data. After comparing all the values, it was noted that for activities like walking and running, maximum correlation coefficient values were found after applying a rotation matrix and activity-wise alignment.

Table 2 shows correlation coefficient values for each activity (multiple walking, running and sitting segments) after applying a rotation matrix and activity-wise alignment. They are listed by clothing type (slacks, skirt and frock) for both waist and thigh sensor data.

From Table 2, the waist sensor data had the highest correlation coefficients, irrespective of clothing type. However, thigh data also showed reasonable correlation values for each activity depending on the clothing type.

### 4.4. Activity Classification

**Figure 6:**
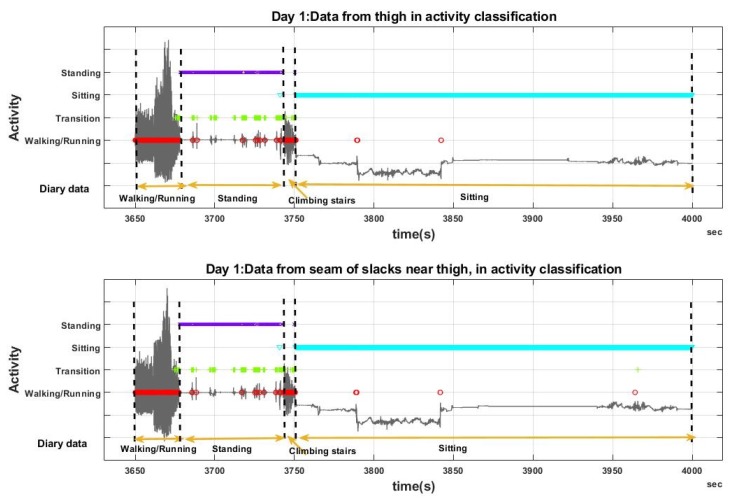
Activity recognition using a decision tree: Day 1 (slacks) data from body worn (top graph) and clothing sensors (bottom graph) were classified into one of four activities i.e., Walking/Running, Transitions of activities, Sitting and Standing. This figure shows a segment of the day’s data. The gravity axis acceleration is plotted in grey, and the outputs of the classifier are denoted in different colours. Red: Walking/Running, Green: Transitions, Cyan: Sitting, Purple: Standing. The participant’s activities according to the diary data are also shown in yellow. The outputs of the classifier are similar in both data files, with minor mismatches.

Figure 6 shows a segment of the output of the activity classifier, based on both body worn and clothing sensors (thigh data on the slacks). This classifier attempted to identify activities i.e., walking/running, transitions, sitting and standing, as denoted on Figure 6. In addition to the classification results, the activities performed by the participant as recorded in the diary are indicated on both graphs.

As the main intention of this research was to examine how the classifier outputs for the clothing sensor data compared with those from the body worn sensor data, and not to calculate the “true” activity classification accuracy, a confusion matrix (Table 3) was created considering the classifications from the body worn data set as the true class. For example, the first cell (row 1, column 1) of Table 3 indicates that 88.0% of the data that was classified as “walking” based on the body worn sensor are also classified as walking based on the clothing worn sensor. Similarly, 9.5% of the data classified as walking based on the body worn sensor are classified as transitions based on the clothing worn sensor.

## 5. Discussion

When using correlation coefficients to compare the data sets, it was important to perform a data alignment for all the sensors, as the correlation was affected by time lags between the sensors’ starting times. Orientation correction at this level is also important as the sensors can become misplaced while the subject is moving and it can mislead the comparisons of data sets. The long term goal is to eliminate the need for time lag and orientation correction by embedding the sensors more effectively in the clothes and engineering synchronous data readings.

The first analysis was done calculating correlation coefficient values for both data files. Table 2 was prepared with a summary of all data from the four common activities that were conducted on three days for waist and thigh sensors. It was clear that thigh data were less correlated than waist sensor data sets. Yet, these values were also significantly correlated with each other. The frock data indicate the possibility of considering clothing dynamics in the sensor data as the frock was a loose dress. Thus the frock could swing with the movements of the leg when the subject was running and walking. Further, when the subject was sitting on a chair, it was noted that the sensor on the clothes near the thigh tended to shift with respect to the sensor worn on the thigh itself. Typically the sensor on the frock would fall away from the leg and onto the chair thus losing a strong relationship to the underlying limb. In addition to the swinging attribute of the frock, the weight of the sensor device (Actigraph) emphasised the movement of the clothing rather than the body. Even though there are no established measures of the looseness of clothes relative to body size, clothing sensor readings would allow these concepts to be explored.

The final analysis was the comparison of the outputs of the activity classifiers. Based on the confusion matrix (Table 3), it was noted that all the activities except the transitions were identified in a high true positive rate, i.e., more than 80%, where the classifier output based on the body-worn sensor was considered as “true”. Hence it can be taken as a positive indication that this would work more accurately when an advanced classifier would be used in activity classification. The findings of [21], mentioned that the accuracy of activity recognition was higher when the sensors were mounted onto clothes. However, they collected the data from a cloth attached to a pendulum. When it comes to data collection from a human with actual clothes, it could be said that our evidence demonstrates a more complex relationship. However, it should be noted that owing to the weight and the size of the Actigraph devices, the correlation of data could have been decreased, and it is better to use smaller, lightweight sensors in a study like this.

When clothing worn sensor data is used for activity classification, it is reasonable to expect that the results will depend on factors that include subject characteristics (e.g., size, gender) as well as clothing styles (looseness, placement). However this study is intended to assess the viability of this approach and hence considers only a single subject across three different clothing types. In future studies, if the sensor positions may vary slightly from day to day due to different positioning of the clothes on the body, this issue can be minimised by rotating the three axis sensor readings along a common axis so as to align the principal direction of gravity with the *y*-axis of the sensor. Moreover, the data distribution for each activity is expected to be the same for *x*, *y* and *z* axis acceleration for sensor readings from different positions. Out of the three types of clothing, the pencil skirt data had the highest correlation as it was the tightest fitting of the clothing used in the study. Moreover, as the clothing waist sensors were more tightly attached to the waist with the clothes, waist sensor data were significantly correlated with each other irrespective of the clothing type.

## 6. Conclusions

This study aimed to assess the suitability of clothing sensor data for use in activity recognition when compared to similarly placed body worn sensors. In this study the clothing sensor data are shown to be well correlated with body worn sensor data as indicated by an analysis of correlation coefficient values. Furthermore the classification results from the clothing sensors are promising when compared to body worn sensors. This is a first study reporting data from sensors embedded into loose clothing in everyday activities. Results indicate that this approach has good potential for daily monitoring, for example in healthcare applications, and that this is an area worthy of further investigation.

This was a single person study intended to gain insight into how data might vary across three different clothing types across a range of likely daily activities. As such the study does not consider benefits of the wide range of different algorithms that could be used for classification. Rather the study checks whether it is possible to collect meaningful data from clothing worn sensors compared to body worn ones. Future studies are now encouraged to improve activity classifiers based on clothing types and supporting the use of multiple lightweight sensors that are networked and time synchronised.

All data used in the paper is available at 10.5281/zenodo.3597391.

## Figures and Tables

**Table 1:** Sensor placement over three days and three types of clothing.

		Day 1	Day 2	Day 3
**Clothes**		Loose slacks	Pencil skirt	Frock (knee-length dress)
**Duration**		5 hours	3 hours	3 hours
**Frequency**		50 Hz	50 Hz	50 Hz
**Sensor** **placement**	**on Body**	Waist	Waist	Waist
		Right thigh	Right thigh	Right thigh
		Right ankle	n/a	n/a
	**on Clothes**	Waist band of slacks	Waist band of skirt	Waist band of frock
		On seam of slacks near thigh	On seam of skirt near thigh	On seam of frock near thigh
		Hem of slacks near ankle	n/a	n/a

**Table 2:** Median correlation coefficient values for different activities for different clothes based on the ‘Waist’ and ‘Thigh’ sensors. Where there were multiple instances of the same activity in a day, the correlation coefficient was calculated for each instance, and the median and variance of the multiple instances is shown. There was a good correlation between body-worn and clothing sensors, apart from the sensor pair on the thigh and seam of the frock.

	Slacks		Skirt		Frock	
	Waist	Thigh	Waist	Thigh	Waist	Thigh
Walking	0.985 ± 0.022	0.945 ± 0.013	0.991 ± 0.006	0.973 ± 0.013	0.978 ± 0.018	0.921 ± 0.059
Running	0.811 ± 0.065	0.802 ± 0.067	0.926 ± 0.0007	0.835 ± 0.094	0.901 ± 0.008	0.642 ± 0.014
Sitting	0.993 ± 0.014	0.967 ± 0.001	0.999 ± 0.0002	0.995 ± 0.004	0.974	0.705
Bus Ride	0.988	0.987	-	-	-	-

**Table 3:** Confusion matrix showing the level to which activity classification based on the clothing sensor data was in agreement with classification based on the body worn sensor data (Day 1 data: when the subject was wearing slacks). Green boxes show when the highest value was expected and also achieved, Yellow boxes indicate where a high value was expected, but a lower value than expected was observed.

	Classification Data from Clothing Worn Sensor against Body Worn Data				
		Walking	Transitions	Sitting	Standing
**Classification Data from the Body** **Worn Sensor as the “True” Class**	**Walking**	88.00%	9.50%	0.70%	1.8%
	**Transitions**	16.10%	45.58%	11.42%	26.90%
	**Sitting**	0.32%	0.26%	88.37%	11.05%
	**Standing**	1.20%	9.58%	0.08%	89.14%
